# Association of brain-derived neurotrophic factor in blood and cerebrospinal fluid with Parkinson’s disease and non-motor symptoms of Parkinson’s disease: a systematic review and meta-analysis of 6655 participants

**DOI:** 10.3389/fnagi.2025.1620172

**Published:** 2025-09-19

**Authors:** Zhenzhen Zhao, Jiahui Sun, Youhong Liu, Miao Liu, Di Tong

**Affiliations:** Department of Geriatrics Center, The Fourth People’s Hospital of Shenyang, Shenyang, China

**Keywords:** brain-derived neurotrophic factor, Parkinson’s disease, non-motor symptom, blood, cerebrospinal fluid, meta-analysis

## Abstract

**Background:**

Brain-derived neurotrophic factor (BDNF) is essential for regulating neuronal proliferation and survival in neurodegenerative diseases, including Parkinson’s disease (PD). However, Studies on BDNF levels in peripheral blood and cerebrospinal fluid (CSF) have inconsistent results. Therefore, this study aimed to examine BDNF levels in patients with PD and to explore their correlation with non-motor symptoms.

**Methods:**

Four databases (PubMed, Embase, Cochrane Library, and CNKI) were searched for eligible studies. The quality of the included studies was assessed using the Newcastle-Ottawa Scale (NOS). Standardized mean differences (SMDs) with 95% confidence intervals (CIs) were calculated using Stata version 14.0, applying either a fixed-effect or a random-effects model based on heterogeneity. Furthermore, subgroup analysis, meta-regression, and sensitivity analysis were employed to identify and analyze sources of heterogeneity. Publication bias was assessed using funnel plots and Egger’s test.

**Results:**

A comprehensive systematic review and meta-analysis was conducted, encompassing 48 articles. A total of 38 studies, covering 2,589 patients with PD and 2,422 healthy controls, were analyzed, revealing significantly lower peripheral blood BDNF levels in PD patients compared to healthy controls (SMD = −1.037; 95% CI [−1.412, −0.662]; *P* < 0.001), with substantial heterogeneity (I^2^ = 97.0%; *P* < 0.001). This result may be more applicable to serum samples and the Asian population according to subgroup analysis. PD patients with depression showed no significant difference in serum BDNF levels compared to those without depression (SMD = −0.511; 95% CI [−1.692, 0.671]; *P* = 0.397). A significant association was found between decreased serum BDNF concentrations and cognitive impairment in PD (SMD = −1.035; 95% CI [−1.340, −0.730]; *P* < 0.001). Moreover, negative correlations were observed between lower serum BDNF levels and autonomic dysfunction, rapid eye movement sleep behavior disorder (RBD), and restless legs syndrome (RLS), respectively. However, CSF BDNF levels showed no statistically significant difference between PD patients and controls (SMD = −0.398; 95% CI [−2.499, 1.703]; *P* = 0.711).

**Conclusion:**

Reduced expression of BDNF is associated with both PD and its non-motor symptoms. Further research is needed to explore the potential of BDNF as a biomarker for non-motor symptoms of PD, particularly for cognitive impairment.

## 1 Introduction

Parkinson’s disease (PD), which is characterized by the deposition of toxic aggregated alpha-synuclein and the loss of dopaminergic neurons, is a complex and chronic neurodegenerative disease. The most frequently observed clinical manifestations among PD patients include bradykinesia and resting tremor, alongside a range of non-motor symptoms such as depression, cognitive impairment, and sleep disturbances. Non-motor symptoms can precede motor symptoms by 10–20 years, placing a significant burden on thousands of families (GBD 2016 Parkinson’s Disease Collaborators, 2018; Leite Silva et al., 2023; Schapira et al., 2017). Depression, affecting approximately 38% of patients with PD, typically emerges early in the disease and persists throughout its course, substantially reducing patients’ quality of life (Cong et al., 2022). The prevalence of cognitive impairment among patients diagnosed with PD is estimated to be as high as 40% (Baiano et al., 2020). However, the significance of non-motor symptoms for the preliminary diagnosis of PD has not been adequately reflected in international diagnostic criteria, highlighting the need for further research to investigate their potential as diagnostic markers for PD.

Brain-derived neurotrophic factor (BDNF), a member of the neurotrophic factor family, is widely distributed in the central nervous system, including astrocytes of the hippocampus and prefrontal cortex. It is also found in non-central nervous system tissues, such as platelets and cardiomyocytes (Palasz et al., 2020). BDNF has a high affinity for tropomyosin receptor kinase B (TrkB) and regulates neuronal maturation and survival–processes that are particularly important in the context of neurodegenerative diseases and psychiatric disorders (Colucci-D’Amato et al., 2020; Numakawa and Kajihara, 2025). Decreased BDNF expression in the striatum has been reported in a PD mouse model induced by 1-methyl-4-phenyl-1,2,3,6-tetrahydropyridine (MPTP) (Zhou et al., 2025). Low expression of BDNF in the medial prefrontal cortex has been observed in a depressive PD rat model, and activation of the BDNF/TrkB pathway can prevent depressive-like behavior in rats (Ma et al., 2024; Tekşen et al., 2024). Furthermore, the *rs6265 BDNF allele* and BDNF serum levels have been linked to various non-motor symptoms in PD (Nikitina et al., 2024a,b).

However, the association of BDNF levels in multiple body fluids with PD remains unclear, with conflicting results reported across studies. A consensus on the correlation between BDNF concentrations and the non-motor symptoms of PD has yet to be reached. Through a systematic review and meta-analysis, the current study aims to investigate BDNF expression in the blood and cerebrospinal fluid (CSF) of PD patients compared to healthy controls. Additionally, it will also explore potential differences between PD patients with and without non-motor symptoms.

## 2 Method

The present meta-analysis was conducted according to the guidelines that are recommended by the PRISMA statement (Preferred Reporting Items for Systematic reviews and Meta-Analysis).

### 2.1 Search strategy and study inclusion

Two investigators (Zhenzhen Zhao and Jiahui Sun) independently searched PubMed, Embase, the Cochrane Library, and CNKI for eligible studies up to March 1, 2025. The search terms were “cerebrospinal fluid,” “serum,” “plasma,” “brain-derived neurotrophic factor,” and “Parkinson’s disease.” The language was restricted to English and Chinese. The inclusion criteria were as follows: (1) case–control study, cross-sectional study, or cohort study that detected BDNF concentrations before experimental intervention; (2) original studies that recorded blood or CSF BDNF concentrations in at least two groups of subjects (PD without non-motor symptoms, PD with non-motor symptoms, and healthy controls); (3) BDNF levels that could be extracted or calculated as mean ± standard deviation from the included studies. The exclusion criteria were as follows: (1) review, report, or letter; (2) samples obtained post-mortem or from animals; (3) necessary data not available or duplicate data reported in different studies. Disagreements between the two investigators regarding study inclusion were resolved by Dr. Liu.

### 2.2 Quality evaluation

The quality of the eligible studies was independently assessed by two investigators using the Newcastle–Ottawa Scale (NOS). Disagreements were resolved by Dr. Liu. The scale evaluates the selection of the study population, the comparability between groups, and the measurement of exposure factors. Scores above 6 were considered high quality.

### 2.3 Data extraction

Data were extracted independently by two investigators, and any discrepancies were resolved through discussion. The extracted data from the eligible studies included the author’s name, year of publication, specimen type, sample size, participants’ age, gender proportion, country, measurement techniques, and BDNF levels. In addition, diagnostic criteria, disease duration, Hoehn–Yahr (H–Y) scores, years of education, and Unified Parkinson’s Disease Rating Scale Part III (UPDRS-III) scores of PD patients were also recorded.

### 2.4 Statistic analysis

Stata version 14.0 software was used to perform the meta-analysis. Standardized mean differences (SMDs) and 95% confidence intervals (CIs) were calculated to compare BDNF levels between groups. The I^2^ statistic and Cochran’s Q test were used to assess heterogeneity between studies. I^2^ values of less than 25%, 50%, and 75% were considered to represent low, moderate, and high heterogeneity, respectively. When significant heterogeneity was present, a random-effects model was applied to pool the data.

Three methods were used to explore the sources of heterogeneity. First, subgroup analysis was performed to assess the effects of specimen type, country of participants, and diagnostic criteria for PD on BDNF levels. Second, sensitivity analysis was conducted to assess the influence of excluding a single study on the overall results by systematically removing one study at a time and reanalyzing the remaining data. Finally, meta-regression was used to examine the effects of sample size, participants’ age, gender proportion, H–Y scores, disease duration, years of education, and UPDRS-III scores of PD patients on the results.

In addition, publication bias was evaluated using funnel plots and Egger’s test. It is worth noting that meta-regression and Egger’s test were not performed when fewer than 10 studies were available. In accordance with standard statistical practice, *P*-values < 0.05 were considered to indicate statistical significance.

## 3 Results

### 3.1 Systematic search and study selection

**FIGURE 1:**
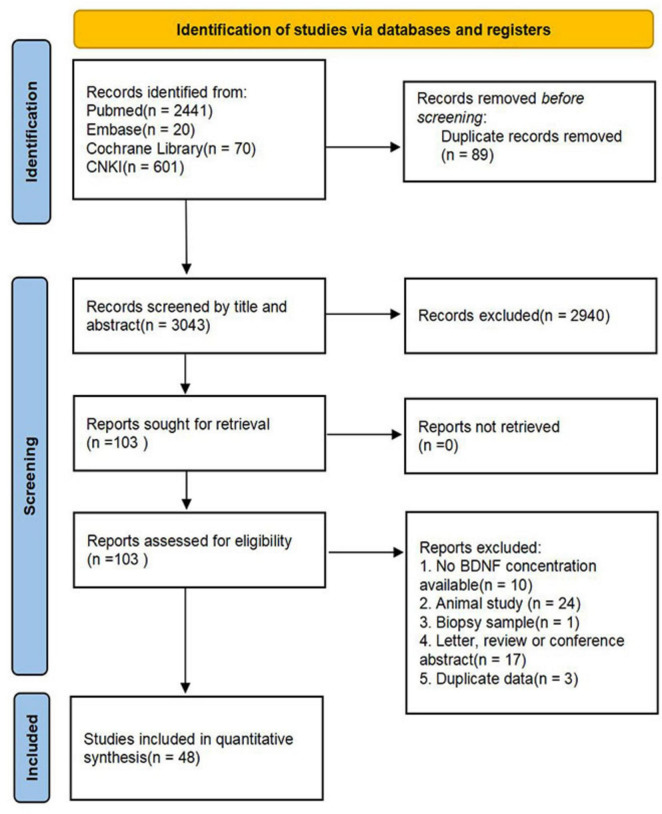
The flowchart summarizes the selection of eligible studies.

The search flowchart was shown in Figure 1. After a comprehensive retrieval and duplicate removal, a total of 3043 potential articles were found through PubMed, Cochrane Library, Embase and CNKI. Following an initial screening of titles and abstracts, 103 articles were deemed eligible for full-text review. Finally, 48 articles that met the inclusion criteria were included in the present systematic review and meta-analysis.

Among the included studies, 38 studies included in the 34 articles reported BDNF levels in the blood of PD patients (*n* = 2,589) and healthy controls (*n* = 2,422) (Alomari et al., 2018; Brockmann et al., 2016; Chen et al., 2015; Costa et al., 2019; Hernández-Vara et al., 2020; Hou et al., 2021; Huang et al., 2018, 2022; Jin et al., 2023; Ju et al., 2018; Khalil et al., 2017; Li and Zhong, n.d.; Li et al., 2019; Liu and Liu, 2023; Peng et al., 2019; Quan et al., 2020; Rocha et al., 2018; Scalzo et al., 2010; Schaeffer et al., 2022; Siuda et al., 2017; Sun, 2011; Szymura et al., 2020; Ventriglia et al., 2013; Wang et al., 2016; Wang and Liu, n.d.; Wu, 2018; Wu et al., 2022; Xie, 2017; Xu et al., 2024; Zhang et al., 2021; Zhao et al., 2021; Zhao and Xu, n.d.; Zhou et al., 2023). Seven studies included in the six articles reported BDNF levels in the blood of PD patients with depression (*n* = 358), PD patients without depression (*n* = 361), and healthy controls (*n* = 290) (Azevedo et al., 2022; Huang et al., 2021a; Ju et al., 2018; Wang et al., 2017, 2024; Wang and Liu, n.d.). Ten studies included in the nine articles examined BDNF levels in the blood of PD patients with cognitive impairment (*n* = 392), PD patients without cognitive impairment (*n* = 346), and healthy controls (*n* = 296) (Hu, 2021; Li and Zhong, n.d.; Li et al., 2019; Lin et al., 2021; Liu et al., 2015; Xiao, 2016; Xie, 2017; Ye et al., 2016; Zhao et al., 2023). Four additional articles explored the relationship between blood BDNF levels and fatigue, autonomic nerve dysfunction, rapid eye movement sleep behavior disorder (RBD), and restless legs syndrome (RLS), respectively (Azevedo et al., 2022; Huang et al., 2021b; Jin et al., 2023; Liu et al., 2024). Additionally, there were three articles noting the BDNF levels in CSF of PD patients (*n* = 78) and controls (*n* = 131) (Pålhagen et al., 2010; Salehi and Mashayekhi, 2009; Zhang et al., 2008). The characteristics of the eligible studies were shown in Supplementary Material.

### 3.2 Blood BDNF levels in PD

**FIGURE 2:**
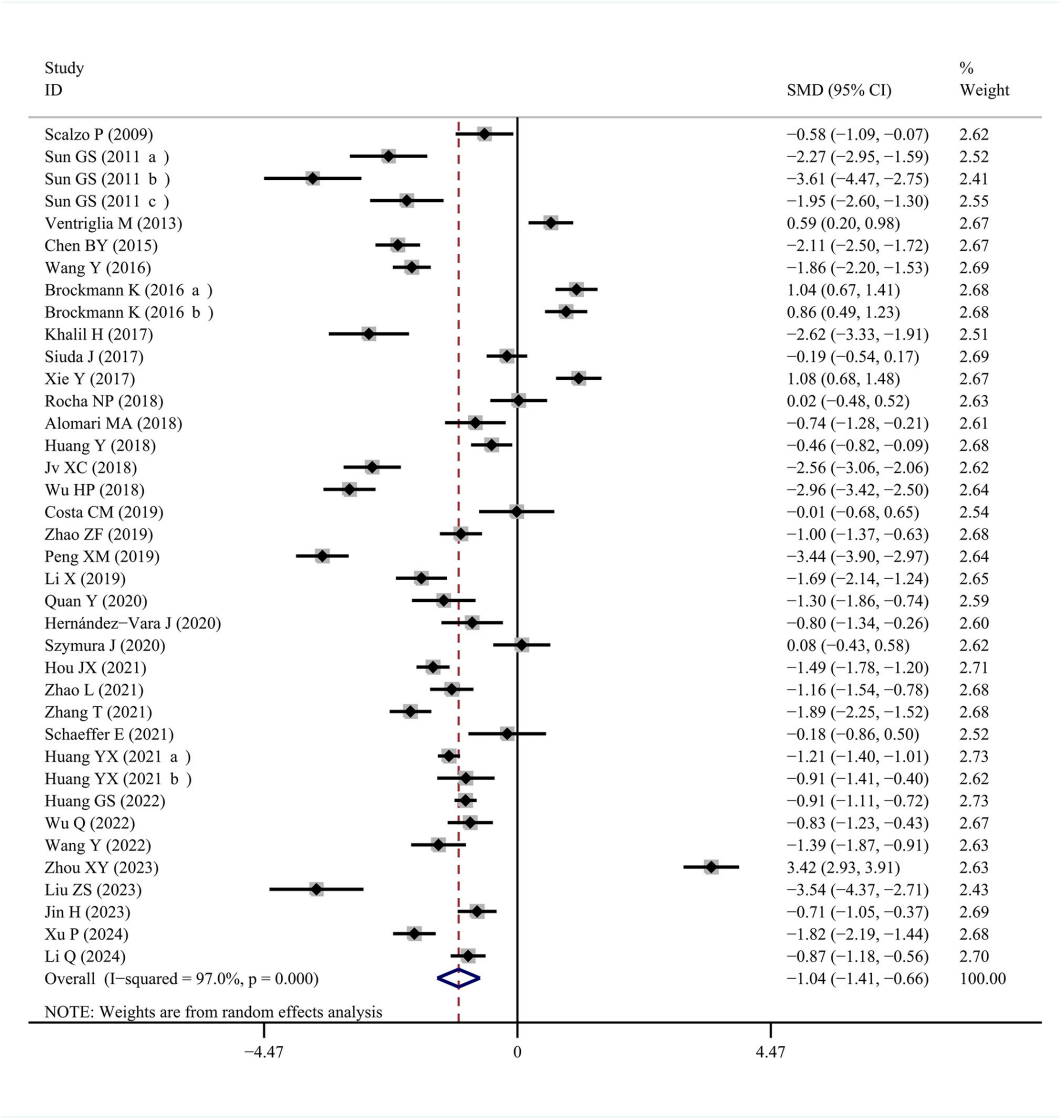
Meta-analysis of studies on blood BDNF in PD patients and controls. CI, confidence interval; SMD, standardized mean difference; BDNF, brain-derived neurotrophic factor; PD, Parkinson’s disease.

Thirty-eight qualified studies reported BDNF levels in blood samples of PD patients compared with healthy controls. Significant heterogeneity was observed among these studies, so a random-effects model was applied (I^2^ = 97.0%, *P* < 0.001). The forest plot showed that BDNF concentrations in blood were significantly lower in PD patients than in healthy controls (SMD = −1.037, 95% CI [−1.412, −0.662], *P* < 0.001). The forest plot was shown in Figure 2.

To explore potential sources of heterogeneity, subgroup analyses were conducted based on specimen type, country, and diagnostic criteria for PD. The results were shown in Table 1. Among the included studies, four used plasma samples, while thirty-four used serum samples. The results showed high heterogeneity in both serum and plasma (plasma: I^2^ = 79.8%, *P* = 0.002; serum: I^2^ = 97.3%, *P* < 0.001). However, the pooled SMD was statistically significant in serum studies (SMD = −1.099, 95% CI [−1.503, −0.694], *P* < 0.001), but not in plasma studies (SMD = −0.513, 95% CI [−1.136, 0.111], *P* = 0.107). These results suggested that the conclusion may only apply to serum.

**TABLE 1:** Subgroup analyses results of BDNF in peripheral blood of PD patients compared with healthy controls.

Factor	Subgroup	No. of studies	Effects model	SMD (95% CI)	*P*	Heterogeneity	
						I^2^ (%)	*P*
Country	Asian	28	Random	−1.436 (−1.846, −1.026)	*P* < 0.001	96.6	*P* < 0.001
	Non-Asian	10	Random	0.103 (−0.287, 0.496)	0.601	86.0	*P* < 0.001
Specimen sample	Serum	34	Random	−1.099 (−1.503, −0.694)	*P* < 0.001	97.3	*P* < 0.001
	Plasma	4	Random	−0.513 (−1.136, 0.111)	0.107	79.8	0.002
PD diagnostic criteria	United Kingdom PD society brain bank criteria	14	Random	−0.259 (−0.895, 0.377)	0.424	97.5	*P* < 0.001
	Other criteria	24	Random	−1.494 (−1.932, −1.055)	*P* < 0.001	96.1	*P* < 0.001

PD, Parkinson’s disease; SMD, standardized mean difference; 95% CI, 95% confidence interval.

Of these studies, twenty-eight were in Asia, while ten were not in Asia. The results showed high heterogeneity in both Asian subgroup and non-Asian subgroup (Asian: I^2^ = 96.8%, *P* < 0.001; non-Asian: I^2^ = 86.0%, *P* < 0.001). A pooled SMD was found to be significant in the Asian subgroup (SMD = −1.436, 95% CI [−1.846, −1.026], *P* < 0.001), but not in the non-Asian subgroup (SMD = 0.105, 95% CI [−0.287, 0.496], *P* = 0.601). These results suggested that the conclusion may only apply to Asian population.

Regarding diagnostic criteria, fourteen studies used the United Kingdom PD Society Brain Bank Criteria (UKPDS), and twenty-four studies used other criteria. The results showed high heterogeneity in both subgroups (UKPDS: I^2^ = 97.5%, *P* < 0.001; other criteria: I^2^ = 96.1%, *P* < 0.001). However, the pooled SMD was significant in other criteria subgroup (SMD = −1.494, 95% CI [−1.932, −1.055], *P* < 0.001), but not in UKPDS subgroup (SMD = −0.259, 95% CI [−0.895, 0.377], *P* = 0.424). All results of subgroup analyses are summarized in Table 1.

In meta-regression analysis, sample sizes, age of participants, sex proportion of participants, H-Y scores, disease duration, year of education and UPDRS-III scores did not show any significant effects on the outcomes. A sensitivity analysis, in which one study was omitted at a time, demonstrated that no single study significantly altered the results.

The application of Egger’s test (*P* > 0.05) and a thorough examination of the funnel plot did not reveal any indication of publication bias.

### 3.3 Serum BDNF levels in PD with depression

**FIGURE 3:**
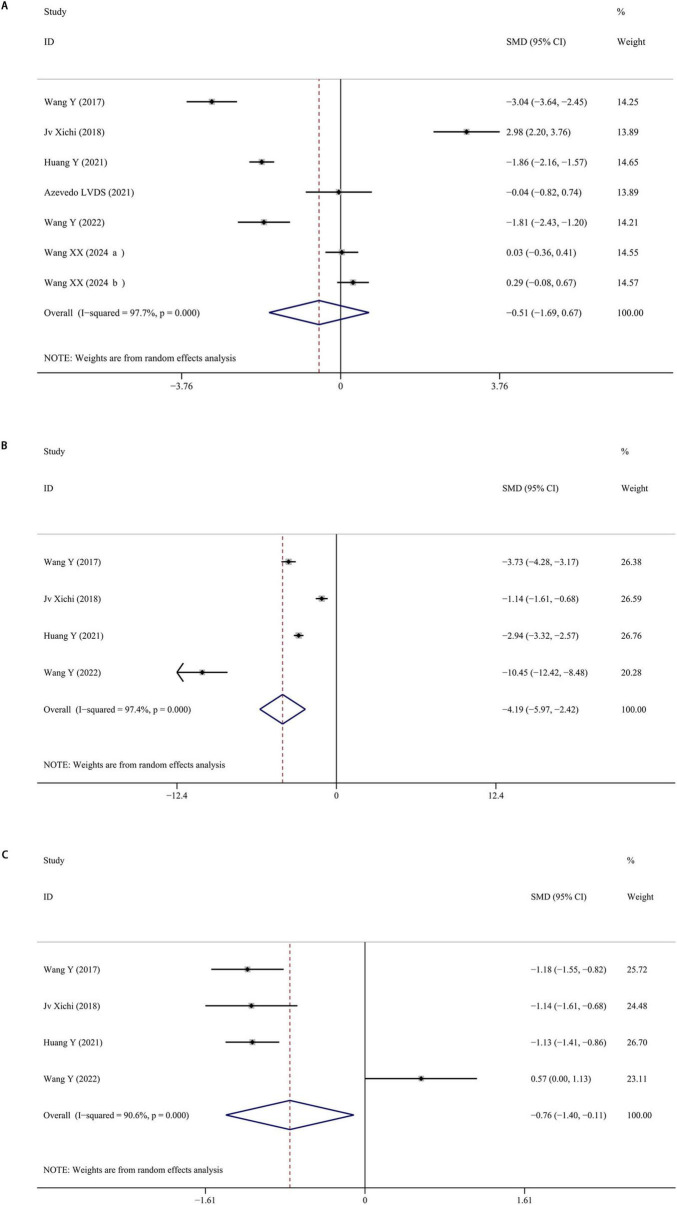
**(A)** Meta-analysis of studies on serum BDNF in PD patients with and without depression. **(B)** Meta-analysis of studies on serum BDNF in PD patients with depression and controls. **(C)** Meta-analysis of studies on serum BDNF in PD patients without depression and controls. CI, confidence interval; SMD, standardized mean difference; BDNF, brain-derived neurotrophic factor; PD, Parkinson’s disease.

Seven studies were included reporting serum BDNF levels in depressed PD patients. The pooled data indicated that BDNF levels were slightly lower in PD patients with depression compared to those without, although this difference was not statistically significant (SMD = −0.511; 95% CI [−1.692, 0.671]; *P* = 0.397), with high heterogeneity (I^2^ = 97.7%; *P* < 0.001). Moreover, there were significantly lower serum BDNF levels in PD with or without depression than healthy controls. The forest plot was shown in Figure 3. Sensitivity analysis indicated that removing one study significantly affected the results, suggesting the instability of the findings. No publication bias was found by visual inspection of the funnel plot.

### 3.4 Serum BDNF levels in PD with cognitive impairment

**FIGURE 4:**
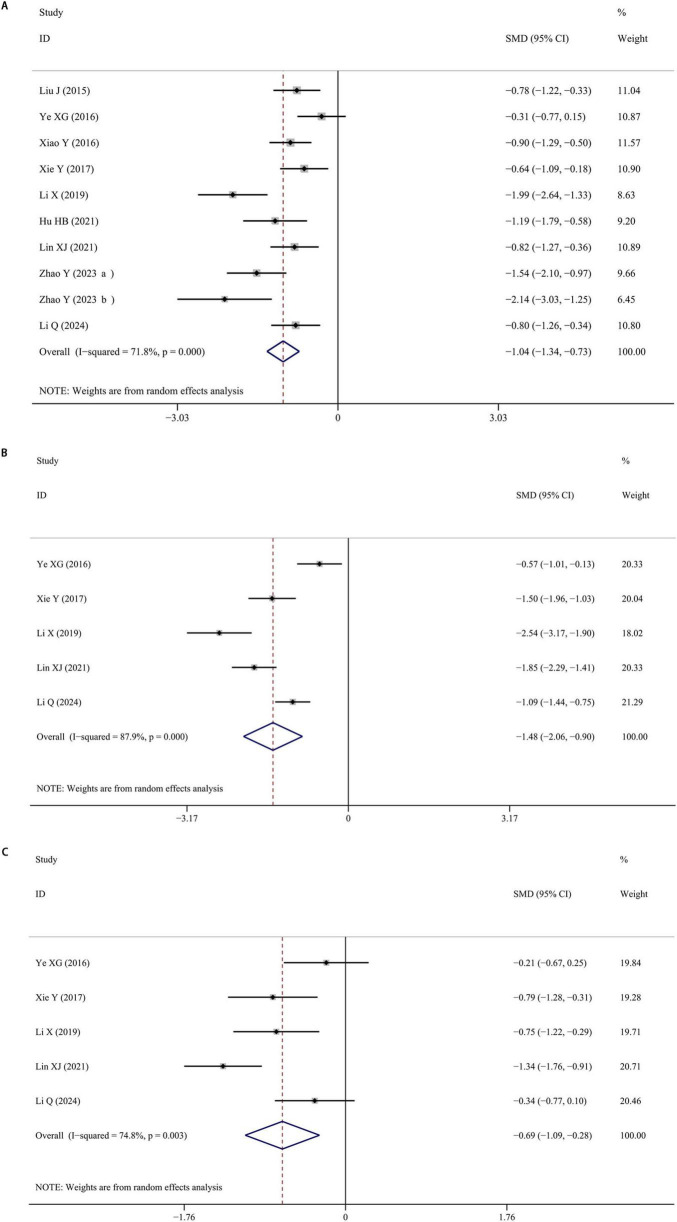
**(A)** Meta-analysis of studies on serum BDNF in PD patients with and without cognitive impairment. **(B)** Meta-analysis of studies on serum BDNF in PD patients with cognitive impairment and controls. **(C)** Meta-analysis of studies on serum BDNF in PD patients without cognitive impairment and controls. CI, confidence interval; SMD, standardized mean difference; BDNF, brain-derived neurotrophic factor; PD, Parkinson’s disease.

Ten studies were included reporting serum BDNF levels of PD patients with cognitive impairment. The pooled data showed that BDNF levels of PD patients with cognitive impairment was decreased than those without cognitive impairment (SMD = −1.035; 95% CI [−1.340, −0.730]; *P* < 0.001), with a high heterogeneity (I^2^ = 71.8%; *P* < 0.001). Furthermore, BDNF levels in patients diagnosed with PD, both with and without cognitive impairment, were notably lower compared to healthy control groups. The forest plot was shown in Figure 4. The results remained stable after omitting one of included studies, demonstrating the stability of the results. The application of Egger’s test (*P* > 0.05) and a thorough examination of the funnel plot did not reveal any indication of publication bias.

### 3.5 Serum BDNF levels in PD with other non-motor symptoms

A study performed in Brazil showed that serum concentrations of BDNF did not significantly differ between PD with fatigue and those without fatigue.

A study performed in China showed that BDNF concentrations of PD with and without autonomic nerve dysfunction were both lower than healthy controls. Furthermore, BDNF levels were significantly lower in PD patients with autonomic nerve dysfunction. This study also revealed a correlation between autonomic nerve dysfunction and H-Y scores through univariate analysis.

A study performed in China indicated that PD patients with RBD had lower BDNF levels than those without RBD. Additionally, BDNF levels were identified as an independent predictor of RBD in PD patients through logistic regression and P-trend analysis.

A study performed in China indicated that PD with RLS had lower BDNF levels than PD without RLS and healthy controls. The study also suggested a correlation between BDNF levels and the severity of RLS.

### 3.6 CSF BDNF levels in PD

**FIGURE 5:**
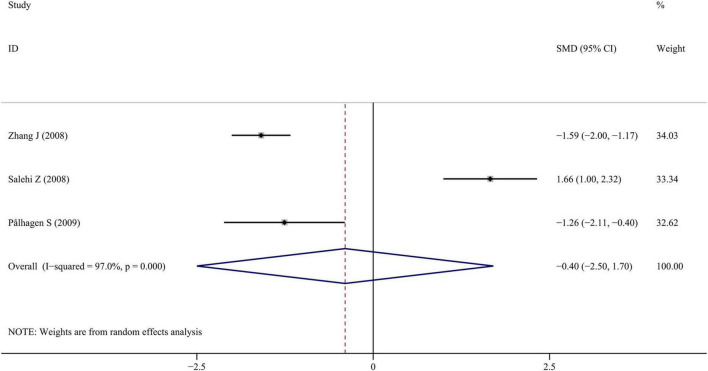
Meta-analysis of studies on CSF BDNF in PD patients and controls. CI, confidence interval; SMD, standardized mean difference; BDNF, brain-derived neurotrophic factor; PD, Parkinson’s disease; CSF, cerebrospinal fluid.

Only three studies reported BDNF levels in the CSF of PD patients. The forest plots from these studies indicated that CSF BDNF levels were slightly lower in PD patients compared to healthy controls, but no significant difference was observed (SMD = −0.398; 95% CI [−2.499, 1.703]; *P* = 0.711). Despite the small number of studies, the results showed high heterogeneity (I^2^ = 97.0%; *P* < 0.001). The forest plot was shown in Figure 5. No publication bias was found by visual inspection of the funnel plot. Sensitivity analysis revealed that the omission of the study by Salehi and Mashayekhi (2009) significantly altered the result, suggesting instability. To draw more reliable conclusions, future studies should increase the sample size.

## 4 Discussion

Although BDNF has been extensively investigated as a key neurotrophic factor in a variety of diseases, no consensus has been reached regarding its expression levels in the blood and CSF of patients with PD, particularly those with non-motor symptoms. To our knowledge, the present study is the first to comprehensively examine the association between blood BDNF levels and non-motor symptoms of PD, including depression, cognitive impairment, sleep disorders, and others. Through the present systematic review and meta-analysis, it was found that BDNF levels in the blood of PD patients were lower than those in healthy controls. Subgroup analyses suggested that these differences might be influenced by specimen type, ethnicity, and diagnostic criteria. However, there was no difference on CSF BDNF levels between PD and healthy controls with limited sample sizes. Additionally, serum BDNF concentrations did not differ significantly between PD patients with and without depression. Lower serum BDNF levels were associated with cognitive impairment, autonomic nerve dysfunction, and sleep disorders in PD patients.

Differences in blood BDNF levels between PD patients and healthy controls observed in our analysis are consistent with previous findings. Two studies demonstrated that serum BDNF was significantly lower in PD patients (Jiang et al., 2019; Rahmani et al., 2019). One study demonstrated that PD patients had decreased BDNF levels than healthy controls in plasma samples (Chen and Zhang, 2023). In our subgroup analysis, the difference was pronounced in serum but sightly in plasma. The physiological concentration of BDNF in CSF and plasma is substantially lower than that in serum under normal conditions (Castrén and Monteggia, 2021). This phenomenon can be explained by the fact that platelets serve as a primary peripheral reservoir for BDNF, and differences in platelet activation between plasma and serum processing can markedly affect peripheral BDNF measurements (Beura et al., 2022; Bouhaddou et al., 2024). Therefore, structural and functional platelet abnormalities reported in PD may contribute to the observed reduction in serum BDNF levels. In contrast, studies of BDNF levels in CSF were significantly constrained by the invasive procedures and the potential risks involved in lumbar puncture. The three included CSF studies in our analysis exhibited marked heterogeneity and yielded inconclusive results, underscoring the need for cautious interpretation and highlighting the importance of larger, standardized investigations in the future.

Of the thirty-eight studies analyzing blood samples, twenty-eight were conducted in Asia, while the remaining ten were primarily from Western Europe. In the non-Asian subgroup, blood BDNF levels in PD patients did not significantly differ from those in healthy controls. It was worth noting that the proportion of plasma studies was higher in non-Asian subgroup, which may partially account for the observed discrepancies between the two subgroups. In a previous meta-analysis, the association between the *BDNF 196 AA* + *AG genotype* and PD was evident in European populations but not in Asian populations, suggesting that interethnic differences in BDNF genotype distribution could influence study outcomes (Lee and Song, 2014). In addition, our analysis indicated that differences in diagnostic criteria may have some effect on the results, although they are unlikely to represent a major source of heterogeneity.

Meta-regression analysis indicated that sample size, age, sex distribution, H–Y scores, disease duration, years of education, and UPDRS-III scores were not significant confounding factors in the present study. However, age, sex proportion and H-Y scores have been identified as confounding factors in previous meta-analyses (Jiang et al., 2019; Rahmani et al., 2019). The discrepancy may be attributed to differences in the number of included studies and the incorporation of Chinese databases in the current analysis. Supporting evidence from individual studies has reported a negative correlation between serum BDNF levels and H–Y scores in PD patients (Huang et al., 2021a). Similarly, a study involving 104 PD patients found inverse associations between BDNF levels and both disease duration and age (Di Lazzaro et al., 2024). Beyond these factors, cognitive performance, motor subtypes, and lifestyle-related variables–such as occupational exposures and caffeine consumption–represent critical variables that warrant detailed consideration and integration in subsequent researches.

Depression, one of the most common non-motor symptoms in PD, is thought to be multi-factorial in etiology. Consistent with previous reports, the present study found no significant difference in serum BDNF levels between PD patients with and without depression (Chen and Zhang, 2023; Rahmani et al., 2019). Nonetheless, several studies have suggested that the *Met allele of BDNF Val66Met polymorphism* has a significantly moderated relationship with depression (Hosang et al., 2014; Zhao et al., 2018; Zou et al., 2024). Lower peripheral BDNF concentrations were found in major depression disease than controls, supporting a potential link between peripheral BDNF expression and depression (Cavaleri et al., 2023; Kishi et al., 2017; Shi et al., 2020; Tiwari et al., 2022). In contrast, other studies have found no clear association between BDNF genotype and depression (Suktas et al., 2024; Wang et al., 2023). Accordingly, the association between BDNF and depression remains undetermined. Moreover, it has been reported that after 7 weeks of antidepressant treatment, BDNF levels in patients with depression become comparable to those in healthy individuals (Gelle et al., 2021). Due to insufficient data, the present analysis could not account for antidepressant use in PD patients with depression, which may partially explain the lack of significant differences observed between depressed and non-depressed PD groups.

Cognitive impairment is among the most frequent non-motor symptoms of PD, though it is often given greater attention in the advanced stages of the disease. Previous studies have demonstrated that lower BDNF levels was associated with cognitive impairment in Alzheimer’s disease and diabetes mellitus (Anita et al., 2022; He et al., 2024; Kim et al., 2017; Xie et al., 2020). Additionally, the *Met allele of BDNF Val66Met polymorphism* has been significantly linked to cognitive impairment in PD (Wang et al., 2019; Yin et al., 2019). These findings collectively support the association between decreased BDNF levels and cognitive impairment of PD in the present analysis. It was noteworthy that the severity of cognitive impairment may influence the relationship (Kim et al., 2017; Ng et al., 2019). Indeed, more severe cognitive decline has been associated with greater reductions in serum BDNF levels (Siuda et al., 2017). Beyond cognitive function, our analysis also identified associations between reduced blood BDNF levels and other non-motor symptoms–including RBD, RLS, and autonomic nerve dysfunction–suggesting that impairment of BDNF-mediated neuroprotective pathways may contribute to both the development of non-motor symptoms and the progression of PD.

In addition to the confounding factors addressed in this study, several other variables–often unreported in the included studies–may also influence the results. Dopamine replacement therapy has been documented to influence BDNF expression, particularly L-3,4-dihydroxyphenylalanine (L-DOPA). In a PD mouse model, repeated L-DOPA administration was observed to increase BDNF levels in the dopamine-depleted subthalamic nucleus (Zhang et al., 2006). Moreover, certain antidepressants, including paroxetine and fluoxetine, have been implicated in altering BDNF expression levels (De Foubert et al., 2004; Martínez-Turrillas et al., 2005). Although the majority of included studies employed the Enzyme-Linked Immunosorbent Assay (ELISA) for measurement, the impact on the resulting data may vary depending on the specific type of reagent and manufacturer utilized. Therefore, standardization of experimental protocols and assay conditions remains essential for generating valid and comparable findings.

## 5 Limitations

There were also some limitations in this study. Firstly, the number of studies included in this analysis is relatively limited, particularly those examining CSF BDNF levels in PD patients. Further investigations are warranted to clarify the relationship between BDNF and non-motor symptoms in PD. Secondly, owing to incomplete or inconsistent reporting in the included studies, several potential sources of heterogeneity could not be fully assessed. Future research should incorporate more comprehensive datasets that include variables such as age at onset, dopamine replacement therapy, and antidepressant use. Additionally, the potential associations between BDNF levels and other non-motor symptoms–such as constipation and olfactory dysfunction–deserve greater attention, as these could contribute to the earlier detection and improved clinical characterization of PD.

## 6 Conclusion

The current study not only examined the concentrations of BDNF in the blood and CSF of PD patients, but also represents the first meta-analysis to characterize trends in BDNF levels among PD patients with non-motor symptoms. We identified a correlation between reduced BDNF expression and a range of non-motor manifestations in PD. These findings provide an evidence-based foundation for future research into the role of BDNF in PD pathogenesis and highlight the importance of further investigating BDNF alterations in PD patients presenting with non-motor symptoms.

## Data Availability

The original contributions presented in this study are included in this article/Supplementary material, further inquiries can be directed to the corresponding author.
