# Supplementary material for: Association of brain-derived neurotrophic factor in blood and cerebrospinal fluid with Parkinson’s disease and non-motor symptoms of Parkinson’s disease: a systematic review and meta-analysis of 6655 participants
Source: Front Aging Neurosci. 2025 Sep 19;17:1620172. doi: 10.3389/fnagi.2025.1620172 (PMC12492986; doi:10.3389/fnagi.2025.1620172)
Supplement: Supplementary file 1 [file Table_1.docx]

Supplementary Table 1. Characteristic of included studies comparing PD patients with healthy controls

| Study | Design | No. of PD | Age of PD | Female proportion of PD | No. of healthy controls | Age of healthy controls | Female proportion of healthy controls | Diagnostic criteria of PD | measurement techniques | Country | Study Quality |
| --- | --- | --- | --- | --- | --- | --- | --- | --- | --- | --- | --- |
| **Serum** | | | | | | | | | | | |
| Ventriglia M,2013 | Case-control | 30 | 67.6±8.4 | 30% | 169 | 48±15.7 | 49% | The Diagnostic and Statistical Manual of Mental Disorders-IV | ELISA | Italy | 8 |
| Scalzo P,2009 | Case-control | 47 | 65.7±8.8 | 49% | 23 | 61.8±10.7 | 65% | Not Mentioned | ELISA | Brazil | 7 |
| Hernández-Vara J,2020 | Case-control | 30 | 63.97 ± 9.59 | 47% | 27 | 47.44 ± 5.99 | 26% | United Kingdom PD Society Brain Bank Criteria | ELISA | Spain | 7 |
| Szymura J,2020 | Case-control | 29 | 65.66 ± 7.48 | 34% | 32 | 66.47 ± 3.21 | 38% | Not Mentioned | ELISA | Poland | 9 |
| Huang Y,2018 | Case-control | 60 | 62.5±9.9 | 47% | 60 | 59.5±14.9 | 52% | United Kingdom PD Society Brain Bank Criteria | ELISA | China | 9 |
| Khalil H,2017 | Case-control | 29 | 59.4±13.1 | 42% | 30 | 56.9±12.8 | 40% | diagnosis of idiopathic Parkinson’s disease confirmed by neurologist’ examination | ELISA | Jordan | 9 |
| Wang Y,2016 | Case-control | 97 | 63.60 ± 9.32 | 44% | 102 | 61.50 ± 9.84 | 43% | United Kingdom PD Society Brain Bank Criteria | ELISA | China | 9 |
| Huang YX,2021(a) | Case-control | 196 | 61.72±9.17 | 48% | 302 | 61.61±9.31 | 44% | United Kingdom PD Society Brain Bank Criteria | ELISA | China | 9 |
| Huang YX,2021(b) | Case-control | 53 | 62.81±9.94 | 57% | 24 | 61.58±13.69 | 54% | United Kingdom PD Society Brain Bank Criteria | ELISA | China | 9 |
| Xu P,2024 | Case-control | 104 | 68.16±8.33 | 60% | 60 | 67.92±8.65 | 55% | MDS clinical diagnostic criteria | ELISA | China | 9 |
| Huang GS, 2022 | Case-control | 253 | 68.83±9.32 | 52% | 200 | 67.26±10.34 | 53% | MDS clinical diagnostic criteria | ELISA | China | 9 |
| Chen BY, 2015 | Case-control | 104 | 74. 3±7. 3 | 48% | 60 | 69. 8 ±4. 7 | 50% | Not Mentioned | ELISA | China | 8 |
| Jv XC,2018 | Case-control | 56 | 65.75±4.9 | 48% | 56 | 65.37±6.88 | 46% | Diagnostic criteria for PD by the Movement Disorders and Parkinson's Disease Group of the Neurology Section of the Chinese Medical Association in 2006 | ELISA | China | 8 |
| Hou JX,2021 | Case-control | 115 | 64.68±5.78 | 49% | 118 | 65.26±4.87 | 43% | Chinese Diagnostic Criteria for Parkinson's Disease in 2016 | ELISA | China | 9 |
| Wu Q,2022 | Case-control | 62 | 64.3 ± 7.6 | 40% | 46 | 64.7 ± 8.4 | 41% | MDS clinical diagnostic criteria | ELISA | China | 9 |
| Zhou XY, 2023 | Case-control | 80 | 64. 59±2. 49 | 56% | 80 | 64. 81±3. 52 | 51% | United Kingdom PD Society Brain Bank Criteria | ELISA | China | 9 |
| Liu ZS,2023 | Case-control | 30 | 66.97±8.15 | 47% | 30 | 66.57±7.80 | 47% | Chinese Diagnostic Criteria for Parkinson's Disease in 2016 | ELISA | China | 9 |
| Brochmann K, 2016(a) | Case-control | 49 | 68(46-83)* | 100% | 89 | 57(18-83)* | 100% | United Kingdom PD Society Brain Bank Criteria | Multiplexed  Immunoassay | Canada,France,Germany,Norway,Spain and the USA | 8 |
| Brochmann K, 2016(b) | Case-control | 95 | 69(46-80)* | 0 | 44 | 58(28-83)* | 0 | United Kingdom PD Society Brain Bank Criteria | Multiplexed  Immunoassay | Canada,France,Germany,Norway,Spain and the USA | 8 |
| Siuda J,2017 | Case-control | 49 | 63.3±10.5 | 45% | 80 | 65.6±11.9 | 55% | United Kingdom PD Society Brain Bank Criteria | ELISA | Poland | 9 |
| Zhao L,2021 | Case-control | 80 | 63.54±9.58 | 43% | 50 | 64.32±8.95 | 46% | Chinese Diagnostic Criteria for Parkinson's Disease in 2016 | ELISA | China | 9 |
| Zhao ZF,2019 | Case-control | 63 | 67.40±7.32 | 48% | 63 | 65.83±11.06 | 52% | Chinese Diagnostic Criteria for Parkinson's Disease in 2016 | ELISA | China | 8 |
| Zhang T,2021 | Case-control | 70 | 62.83±8.23 | 54% | 100 | 63.12±8.94 | 55% | Chinese Diagnostic Criteria for Parkinson's Disease in 2016 | ELISA | China | 9 |
| Peng XM,2019 | Case-control | 100 | 57.41±7.93 | 43% | 80 | 57.19±7.45 | 46% | Chinese Diagnostic Criteria for Parkinson's Disease in 2016 | ELISA | China | 9 |
| Wu HP,2018 | Case-control | 60 | 62-77 | 47% | 100 | 61-79 | 39% | Chinese Diagnostic Criteria for Parkinson's Disease in 2016 | ELISA | China | 8 |
| Li Q,2024 | Case-control | 93 | 69.76±10.23 | 51% | 86 | 66.51±11.18 | 55% | United Kingdom PD Society Brain Bank Criteria | ELISA | China | 8 |
| Xie Y,2017 | Case-control | 84 | 67.7±9.4 | 48% | 40 | 67.7±9.4 | 45% | Chinese Diagnostic Criteria for Parkinson's Disease in 2016 | ELISA | China | 8 |
| Wang Y,2022 | Case-control | 62 | 66.02±7.69 | 53% | 30 | 65.97±8.88 | 50% | Chinese Diagnostic Criteria for Parkinson's Disease in 2016 | ELISA | China | 9 |
| Li X,2019 | Case-control | 55 | 66.76±7.52 | 40% | 50 | 67.01±7.89 | 42% | Chinese Diagnostic Criteria for Parkinson's Disease in 2006 | ELISA | China | 8 |
| Sun GS,2011(a) | Case-control | 40 | 55.62±6.47 | 50% | 20 | 58.11±5.97 | 50% | Chinese Diagnostic Criteria for Parkinson's Disease in 2006 | Not Mentioned | China | 8 |
| Sun GS,2011(b) | Case-control | 40 | 57.13±7.29 | 50% | 20 | 58.11±5.97 | 50% | Chinese Diagnostic Criteria for Parkinson's Disease in 2006 | Not Mentioned | China | 8 |
| Sun GS,2011(c) | Case-control | 40 | 61.67±7.15 | 50% | 20 | 58.11±5.97 | 50% | Chinese Diagnostic Criteria for Parkinson's Disease in 2006 | Not Mentioned | China | 8 |
| Jin H,2023 | Case-control | 101 | 60.82±10.30 | 44% | 53 | 62.85±10.34 | 42% | MDS clinical diagnostic criteria | ELISA | China | 9 |
| Schaeffer E,2021 | Case-control | 17 | 58±10 | 47% | 16 | 58±12 | 50% | United Kingdom PD Society Brain Bank Criteria | ELISA | Germany | 9 |
| **Plasma** | | | | | | | | | | | |
| Quan Y,2020 | Case-control | 30 | 67.19 ± 8.12 | 43% | 30 | 68.63 ± 7.17 | 50% | United Kingdom PD Society Brain Bank Criteria | ELISA | China | 8 |
| Costa CM,2019 | Case-control | 18 | 68(62.5–71.5)** | 33% | 17 | 62 (60–66)** | 41% | Not Mentioned | ELISA | Brazil | 8 |
| Rocha NP,2018 | Case-control | 40 | 68.71±10.07 | 33% | 25 | 65.23±8.75 | 24% | United Kingdom PD Society Brain Bank Criteria | ELISA | Brazil | 9 |
| Alomari MA,2018 | Case-control | 28 | 59.4 ± 13.1 | NA | 30 | 56.9± 12.8 | NA | Not Mentioned | ELISA | Jordan | 7 |
| **CSF** | | | | | | | | | | | |
| Pålhagen S,2009 | Case-control | 14 | 65.3±7.2 | 43% | 12 | 29.4 ± 1.2 | NA | United Kingdom PD Society Brain Bank Criteria | Enzyme Immunoassays(EIAs) | Sweden | 8 |
| Zhang J,2008 | Case-control | 40 | 59 ± 10 | 23% | 95 | 63 ± 12 | 54% | Not Mentioned | immunobead-based multiplex assays | USA | 8 |
| Salehi Z,2008 | Case-control | 24 | NA | NA | 24 | NA | NA | Not Mentioned | ELISA | Iran | 5 |

PD, Parkinson’s disease; ELISA, Enzyme-linked immunosorbent assay; NA, Not available; USA, The United States of American

*:median(range); **:median(IQ range); Other data were presented as mean±standard deviation

Supplementary Table 2. Characteristic of included studies about PD patients with non-motor symptoms

| Study | Specimen Type | No. of PD with Non-motor symptoms | Age of PD with Non-motor symptoms | Female proportion of PD with Non-motor symptoms | No. of PD without Non-motor symptoms | Age of PD without Non-motor symptoms | Female proportion of PD without Non-motor symptoms | No. of healthy controls | Age of healthy controls | Female proportion of healthy controls | Diagnostic criteria of PD | Country | Study Quality |
| --- | --- | --- | --- | --- | --- | --- | --- | --- | --- | --- | --- | --- | --- |
| **Depression** | | | | | | | | | | | | | |
| Huang Y,2021 | Serum | 122 | 62.84±8.73 | 48% | 137 | 62.19±9.51 | 46% | 110 | 62.58±10.87 | 44% | United Kingdom PD Society Brain Bank Criteria | China | 9 |
| Wang Y,2017 | Serum | 46 | 63.85±9.50 | 48% | 50 | 61.64±8.87 | 38% | 102 | 61.50±9.84 | 43% | United Kingdom PD Society Brain Bank Criteria | China | 9 |
| Azevedo LVDS,2021 | Serum | 9 | 60.6 ± 9.0 | 0 | 21 | 65.2 ± 10.1 | 29% | NA | NA | NA | United Kingdom PD Society Brain Bank Criteria | Brazil | 8 |
| Jv XC,2018 | Serum | 23 | NA | NA | 33 | NA | NA | 56 | 65.37±6.88 | 46% | Chinese Diagnostic Criteria for Parkinson's Disease in 2006 | China | 8 |
| Wang Y,2022 | Serum | 40 | 67.22±8.31 | 58% | 22 | 63.82±7.97 | 46% | 30 | 65.97±8.88 | 50% | Chinese Diagnostic Criteria for Parkinson's Disease in 2016 | China | 9 |
| Wang XX,2024(a) | Serum | 46 | 67.52±7.00 | 0 | 58 | 64.31±6.66 | 0 | NA | NA | NA | Clinical Diagnostic Criteria for Movement Disorder Society | China | 8 |
| Wang XX,2024(b) | Serum | 62 | 65.56±7.07 | 100% | 50 | 65.32±6.24 | 100% | NA | NA | NA | Clinical Diagnostic Criteria for Movement Disorder Society | China | 8 |
| **Cognitive Impairment** | | | | | | | | | | | | | |
| Ye XG,2016 | Serum | 43 | 67.6±8.5 | 47% | 33 | 65.2±9.2 | 45% | 40 | 66.2±8.4 | 45% | United Kingdom PD Society Brain Bank Criteria | China | 9 |
| Li Q,2021 | Serum | 66 | 71.18±9.13 | 50% | 27 | 66.30±12.02 | 52% | 86 | 66.51±11.18 | 55% | United Kingdom PD Society Brain Bank Criteria | China | 9 |
| Hu HB,2021 | Serum | 21 | NA | NA | 31 | NA | NA | NA | NA | NA | Clinical Diagnostic Criteria for Movement Disorder Society | China | 6 |
| Zhao Y,2023 | Serum | 33 | 67.43±6.89 | 42% | 31 | 65.97±7.36 | 52% | NA | NA | NA | Clinical Diagnostic Criteria for Movement Disorder Society | China | 8 |
| Liu XJ,2021 | Serum | 43 | 65.1±6.4 | 47% | 38 | 65.4±6.9 | 47% | 80 | 66.1±6.5 | 0.48 | Chinese Diagnostic Criteria for Parkinson's Disease in 2016 | China | 9 |
| Xie Y,2017 | Serum | 53 | NA | NA | 31 | NA | NA | 40 | 67. 7±9. 4 | 45% | Chinese Diagnostic Criteria for Parkinson's Disease in 2016 | China | 8 |
| Liu J,2015 | Serum | 45 | 65. 7±4. 3 | 51% | 40 | 65. 2±4. 6 | 58% | NA | NA | NA | Chinese Diagnostic Criteria for Parkinson's Disease in 2016 | China | 8 |
| Xiao Y,2016 | Serum | 54 | 65.8±4.3 | 48% | 54 | 66.2±4.0 | 44% | NA | NA | NA | Chinese Diagnostic Criteria for Parkinson's Disease in 2016 | China | 8 |
| Li X,2019 | Serum | 25 | NA | NA | 30 | NA | NA | 50 | 67.01±7.89 | 42% | Chinese Diagnostic Criteria for Parkinson's Disease in 2016 | China | 8 |
| **Fatigue** | | | | | | | | | | | | | |
| Azevedo LVDS,2021 | Serum | 9 | 63.6 ± 9.4 | 11% | 21 | 64.0 ± 10.3 | 24% | NA | NA | NA | United Kingdom PD Society Brain Bank Criteria | Brazil | 8 |
| **autonomic nerve dysfunction** | | | | | | | | | | | | | |
| Liu LJ,2024 | Serum | 63 | 67.21±5.27 | 44% | 125 | 66.92±4.65 | 46% | 140 | NA | NA | Chinese Diagnostic Criteria for Parkinson's Disease in 2016 | China | 8 |
| **RBD** | | | | | | | | | | | | | |
| Jin H，2023 | Serum | 45 | 63 (59, 69)** | 42% | 56 | 58.5 (52.5, 65)** | 45% | 53 | 62.85±10.34 | 42% | MDS clinical diagnostic criteria | China | 9 |
| **RLS** | | | | | | | | | | | | | |
| Huang YX，2021 | Serum | 53 | 62.81±9.94 | 57% | 196 | 61.72±9.17 | 48% | 302 | 61.61±9.31 | 44% | United Kingdom PD Society Brain Bank Criteria | China | 9 |

PD, Parkinson’s disease; NA, Not available; RBD, Rapid Eye Movement Sleep Behavior Disorder; RLS, Restless Legs Syndrome

**:median(IQ range); Other data were presented as mean±standard deviation

Supplementary Table 3. Quality Assessment of Included Studies by Newcastle-Ottawa Scale

| Study | Selection | | | | Comparability | Exposure | | | Total Scores |
| --- | --- | --- | --- | --- | --- | --- | --- | --- | --- |
|  | Is the case definition adequate? | Representativeness of the cases | Selection of Controls | Definition of Controls | Comparability of cases and controls on the basis of the design or analysis | Ascertainment of exposure | Same method of ascertainment for cases and controls | Non-Response rate |  |
| **Serum studies（Parkinson’s disease vs healthy controls）** | | | | | | | | | |
| Ventriglia M,2013 | ⭐ | ⭐ | ⭐ | ⭐ | ⭐（Age discrepancy） | ⭐ | ⭐ | ⭐ | 8 |
| Scalzo P,2009 | yes, but specific diagnostic criteria were not mentioned | ⭐ | no description | ⭐ | ⭐⭐ | ⭐ | ⭐ | ⭐ | 7 |
| Hernández-Vara J,2020 | ⭐ | ⭐ | Hospital control | ⭐ | ⭐（Age discrepancy） | ⭐ | ⭐ | ⭐ | 7 |
| Szymura J,2020 | ⭐ | ⭐ | ⭐ | ⭐ | ⭐⭐ | ⭐ | ⭐ | ⭐ | 9 |
| Huang Y,2018 | ⭐ | ⭐ | ⭐ | ⭐ | ⭐⭐ | ⭐ | ⭐ | ⭐ | 9 |
| Khalil H,2017 | ⭐ | ⭐ | ⭐ | ⭐ | ⭐⭐ | ⭐ | ⭐ | ⭐ | 9 |
| Wang Y,2016 | ⭐ | ⭐ | ⭐ | ⭐ | ⭐⭐ | ⭐ | ⭐ | ⭐ | 9 |
| Huang YX,2021(a) | ⭐ | ⭐ | ⭐ | ⭐ | ⭐⭐ | ⭐ | ⭐ | ⭐ | 9 |
| Huang YX,2021(b) | ⭐ | ⭐ | ⭐ | ⭐ | ⭐⭐ | ⭐ | ⭐ | ⭐ | 9 |
| Xu P,2024 | ⭐ | ⭐ | ⭐ | ⭐ | ⭐⭐ | ⭐ | ⭐ | ⭐ | 9 |
| Huang GS, 2022 | ⭐ | ⭐ | ⭐ | ⭐ | ⭐⭐ | ⭐ | ⭐ | ⭐ | 9 |
| Chen BY, 2015 | yes, but specific diagnostic criteria were not mentioned | ⭐ | ⭐ | ⭐ | ⭐⭐ | ⭐ | ⭐ | ⭐ | 8 |
| Jv XC,2018 | ⭐ | ⭐ | Hospital control | ⭐ | ⭐⭐ | ⭐ | ⭐ | ⭐ | 8 |
| Hou JX,2021 | ⭐ | ⭐ | ⭐ | ⭐ | ⭐⭐ | ⭐ | ⭐ | ⭐ | 9 |
| Wu Q,2022 | ⭐ | ⭐ | ⭐ | ⭐ | ⭐⭐ | ⭐ | ⭐ | ⭐ | 9 |
| Zhou XY, 2023 | ⭐ | ⭐ | ⭐ | ⭐ | ⭐⭐ | ⭐ | ⭐ | ⭐ | 9 |
| Liu ZS,2023 | ⭐ | ⭐ | ⭐ | ⭐ | ⭐⭐ | ⭐ | ⭐ | ⭐ | 9 |
| Brochmann K, 2016(a) | ⭐ | ⭐ | no description | ⭐ | ⭐⭐ | ⭐ | ⭐ | ⭐ | 8 |
| Brochmann K, 2016(b) | ⭐ | ⭐ | no description | ⭐ | ⭐⭐ | ⭐ | ⭐ | ⭐ | 8 |
| Siuda J,2017 | ⭐ | ⭐ | ⭐ | ⭐ | ⭐⭐ | ⭐ | ⭐ | ⭐ | 9 |
| Zhao L,2021 | ⭐ | ⭐ | ⭐ | ⭐ | ⭐⭐ | ⭐ | ⭐ | ⭐ | 9 |
| Zhao ZF,2019 | ⭐ | ⭐ | no description | ⭐ | ⭐⭐ | ⭐ | ⭐ | ⭐ | 8 |
| Zhang T,2021 | ⭐ | ⭐ | ⭐ | ⭐ | ⭐⭐ | ⭐ | ⭐ | ⭐ | 9 |
| Peng XM,2019 | ⭐ | ⭐ | ⭐ | ⭐ | ⭐⭐ | ⭐ | ⭐ | ⭐ | 9 |
| Wu HP,2018 | yes, but specific diagnostic criteria were not mentioned | ⭐ | ⭐ | ⭐ | ⭐⭐ | ⭐ | ⭐ | ⭐ | 8 |
| Li Q,2024 | ⭐ | ⭐ | ⭐ | ⭐ | ⭐（Age discrepancy） | ⭐ | ⭐ | ⭐ | 8 |
| Xie Y,2017 | ⭐ | ⭐ | Hospital control | ⭐ | ⭐⭐ | ⭐ | ⭐ | ⭐ | 8 |
| Wang Y,2022 | ⭐ | ⭐ | ⭐ | ⭐ | ⭐⭐ | ⭐ | ⭐ | ⭐ | 9 |
| Li X,2019 | ⭐ | ⭐ | Hospital control | ⭐ | ⭐⭐ | ⭐ | ⭐ | ⭐ | 8 |
| Sun GS,2011(a) | ⭐ | ⭐ | Hospital control | ⭐ | ⭐⭐ | ⭐ | ⭐ | ⭐ | 8 |
| Sun GS,2011(b) | ⭐ | ⭐ | Hospital control | ⭐ | ⭐⭐ | ⭐ | ⭐ | ⭐ | 8 |
| Sun GS,2011(c) | ⭐ | ⭐ | Hospital control | ⭐ | ⭐⭐ | ⭐ | ⭐ | ⭐ | 8 |
| Jin H,2023 | ⭐ | ⭐ | ⭐ | ⭐ | ⭐⭐ | ⭐ | ⭐ | ⭐ | 9 |
| Schaeffer E,2021 | ⭐ | ⭐ | ⭐ | ⭐ | ⭐⭐ | ⭐ | ⭐ | ⭐ | 9 |
| **Plasma studies（Parkinson’s disease vs healthy controls）** | | | | | | | | | |
| Quan Y,2020 | ⭐ | ⭐ | no description | ⭐ | ⭐⭐ | ⭐ | ⭐ | ⭐ | 8 |
| Costa CM,2019 | ⭐ | ⭐ | ⭐ | ⭐ | ⭐（gender discrepancy） | ⭐ | ⭐ | ⭐ | 8 |
| Rocha NP,2018 | ⭐ | ⭐ | ⭐ | ⭐ | ⭐⭐ | ⭐ | ⭐ | ⭐ | 9 |
| Alomari MA,2018 | yes, but specific diagnostic criteria were not mentioned | ⭐ | ⭐ | ⭐ | ⭐（gender discrepancy） | ⭐ | ⭐ | ⭐ | 7 |
| **Cerebrospinal fluid studies（Parkinson’s disease vs healthy controls）** | | | | | | | | | |
| Pålhagen S,2009 | yes, but specific diagnostic criteria were not mentioned | ⭐ | ⭐ | ⭐ | ⭐⭐ | ⭐ | ⭐ | ⭐ | 8 |
| Zhang J,2008 | ⭐ | ⭐ | ⭐ | ⭐ | ⭐（gender discrepancy） | ⭐ | ⭐ | ⭐ | 8 |
| Salehi Z,2008 | no description | ⭐ | no description | ⭐ | no description | ⭐ | ⭐ | ⭐ | 5 |
| **Serum studies（Parkinson’s disease with depression vs Parkinson’s disease without depression）** | | | | | | | | | |
| Huang Y,2021 | ⭐ | ⭐ | ⭐ | ⭐ | ⭐⭐ | ⭐ | ⭐ | ⭐ | 9 |
| Wang Y,2017 | ⭐ | ⭐ | ⭐ | ⭐ | ⭐⭐ | ⭐ | ⭐ | ⭐ | 9 |
| Azevedo LVDS,2021 | ⭐ | ⭐ | ⭐ | ⭐ | ⭐（gender discrepancy） | ⭐ | ⭐ | ⭐ | 8 |
| Jv XC,2018 | ⭐ | ⭐ | Hospital control | ⭐ | ⭐⭐ | ⭐ | ⭐ | ⭐ | 8 |
| Wang Y,2022 | ⭐ | ⭐ | ⭐ | ⭐ | ⭐⭐ | ⭐ | ⭐ | ⭐ | 9 |
| Wang XX,2024(a) | ⭐ | ⭐ | Hospital control | ⭐ | ⭐⭐ | ⭐ | ⭐ | ⭐ | 8 |
| Wang XX,2024(b) | ⭐ | ⭐ | Hospital control | ⭐ | ⭐⭐ | ⭐ | ⭐ | ⭐ | 8 |
| **Serum studies（Parkinson’s disease with cognitive impairment vs Parkinson’s disease without cognitive impairment）** | | | | | | | | | |
| Ye XG,2016 | ⭐ | ⭐ | ⭐ | ⭐ | ⭐⭐ | ⭐ | ⭐ | ⭐ | 9 |
| Li Q,2021 | ⭐ | ⭐ | ⭐ | ⭐ | ⭐⭐ | ⭐ | ⭐ | ⭐ | 9 |
| Hu HB,2021 | ⭐ | ⭐ | Hospital control | ⭐ | no description | ⭐ | ⭐ | ⭐ | 6 |
| Zhao Y,2023 | ⭐ | ⭐ | Hospital control | ⭐ | ⭐⭐ | ⭐ | ⭐ | ⭐ | 8 |
| Liu XJ,2021 | ⭐ | ⭐ | ⭐ | ⭐ | ⭐⭐ | ⭐ | ⭐ | ⭐ | 9 |
| Xie Y,2017 | ⭐ | ⭐ | Hospital control | ⭐ | ⭐⭐ | ⭐ | ⭐ | ⭐ | 8 |
| Liu J,2015 | ⭐ | ⭐ | Hospital control | ⭐ | ⭐⭐ | ⭐ | ⭐ | ⭐ | 8 |
| Xiao Y,2016 | ⭐ | ⭐ | Hospital control | ⭐ | ⭐⭐ | ⭐ | ⭐ | ⭐ | 8 |
| Li X,2019 | ⭐ | ⭐ | Hospital control | ⭐ | ⭐⭐ | ⭐ | ⭐ | ⭐ | 8 |
| **Serum studies（Parkinson’s disease with other non-motor symptoms vs Parkinson’s disease without other non-motor symptoms）** | | | | | | | | | |
| Azevedo LVDS,2021 | ⭐ | ⭐ | ⭐ | ⭐ | ⭐（gender discrepancy） | ⭐ | ⭐ | ⭐ | 8 |
| Liu LJ,2024 | ⭐ | ⭐ | Hospital control | ⭐ | ⭐⭐ | ⭐ | ⭐ | ⭐ | 8 |
| Jin H，2023 | ⭐ | ⭐ | ⭐ | ⭐ | ⭐⭐ | ⭐ | ⭐ | ⭐ | 9 |
| Huang YX，2021 | ⭐ | ⭐ | ⭐ | ⭐ | ⭐⭐ | ⭐ | ⭐ | ⭐ | 9 |
